# Mobile application to support oncology patients during treatment on patient outcomes: Evidence from a randomized controlled trial

**DOI:** 10.1002/cam4.5351

**Published:** 2022-10-18

**Authors:** Ilana Graetz, Xin Hu, Andrea N. Curry, Andrew Robles, Gregory A. Vidal, Lee S. Schwartzberg

**Affiliations:** ^1^ Department of Health Policy and Management Rollins School of Public Health, Emory University Atlanta Georgia USA; ^2^ West Cancer Center and Research Institute Germantown Tennessee USA; ^3^ Renown Institute for Cancer Reno Nevada USA

**Keywords:** cancer treatment, chemotherapy, mobile health, randomized control trial, self‐management support

## Abstract

**Background:**

Cancer treatment requires substantial demands on patients and their caregivers. Mobile apps can provide support for self‐management during oncology treatment, but few have been rigorously evaluated.

**Methods:**

A 3‐month randomized controlled trial was conducted at a large cancer center to evaluate the efficacy of an app (LivingWith®) that provides self‐management support during cancer treatment on quality of life and health care utilization. Patients in chemotherapy treatment were randomized into the intervention (*n* = 113) and control group (*n* = 111). Intervention group participants agreed to use the app weekly for 3 months, and all participants completed a survey at enrollment and after 3 months to evaluate changes in quality of life and health care utilization.

**Results:**

Retention rate was 75.4% with 169 participants completing the follow‐up survey. The intervention group reported 0.74 fewer medical office visits (*p* = 0.043) and 0.24 fewer visits with a mental health professional (*p* = 0.061) during the 3 and month intervention compared with controls. There were no significant changes by study group in quality of life, or emergency room and urgent care visits. Among intervention participants, 75.3% reported using the app and on average, used it 11.7 times during the 3‐month intervention. Reasons for not using the app among intervention participants included lack of time, lack of interest in apps, and usability challenges.

**Conclusions and Relevance:**

Apps are inexpensive and scalable tools that can provide additional support for individuals coping with complex cancer treatments. This trial provides evidence that a well‐designed oncology support app used during chemotherapy resulted in fewer clinic visits. Still, nearly a quarter of participants randomized to the intervention arm reported never using the app due to personal preference and usability challenges, which points to future opportunities for calibrating target user population and improving user‐centered design.

Clinicaltrials.gov identifier: NCT04331678.

## Abstract 2

This randomized controlled trial of oncology patients provides evidence that a well‐designed oncology support mobile app used during chemotherapy resulted in fewer clinic visits.

## 1 INTRODUCTION

Cancer treatments, especially chemotherapy, often require substantial demands on patients and their informal caregivers, including managing an overwhelming amount of information to coordinate treatment and related activities. Comprehensive coordinating efforts are needed to manage chemotherapy treatments that usually include a complex schedule and are associated with significant or even disabling side effects. Additionally, diagnostic testing and other therapeutic modalities (e.g., surgery and radiation) may require multiple visits with different providers at various locations.

The increasing penetration of smartphones across socioeconomic groups in the United States can be leveraged by individuals dealing with cancer to support their efforts to manage their health, coordinate appointments, and keep track of treatment notes and questions.^1^, ^2^, ^3^ Leveraging mobile health technologies to improve patient health care access and engagement has been proposed by the Institute of Medicine and the American Society of Clinical Oncology as a method of decreasing medical errors and increasing health care quality.^4^, ^5^, ^6^ Although thousands of apps are available to support patients during treatment, rigorous evidence is needed to understand the advantages and limitations of this technology.^3^ Prior research found that although hundreds of apps are available for cancer patients to download, few provide support for self‐management activities, and even fewer have been rigorously evaluated.^7^, ^8^, ^9^, ^10^

We designed this randomized controlled trial to evaluate the usability of a patient‐facing application (app) and test its impact on patients' quality of life and health care utilization. The app, LivingWith®, was developed by Pfizer to support individuals undergoing cancer treatment. Changes in these endpoints were evaluated from baseline to 3 months after the intervention relative to the control group.

## 2 METHODS

The study protocol was approved by the University of Tennessee Health Science Center Institutional Review Board and was registered with ClinicalTrials.gov (NCT04331678).

### 2.1 Participants

This randomized controlled trial was conducted at West Cancer Center and Research Institute (WCCRI) between June 2020 and December 2021. WCCRI is a large comprehensive cancer center with nine clinics and over 70 physicians serving 60% of all patients in the tri‐state area of west Tennessee, north Mississippi, and east Arkansas. The study site is an industry leader in implementing innovative technology to support patient engagement activities aimed at improving patient outcomes. To be eligible, WCCRI patients had to be ≥18 years old, with a confirmed cancer diagnosis within 1 month of chemotherapy initiation, had a valid email address, a smartphone device with a data plan, and were willing to download and use the app. Patients unable to communicate in English were excluded, as only English versions of the surveys and app were available at the time.

### 2.2 Recruitment and randomization

Potentially eligible patients with a diagnosis of active solid tumor or hematologic malignancy were identified for recruitment by a research staff member using the WCCRI's electronic health record system. After confirming eligibility and completing informed consent, participants completed the enrollment survey and were randomly assigned in a 1:1 ratio to one of the two study groups: intervention and control.

### 2.3 Intervention

**FIGURE 1:**
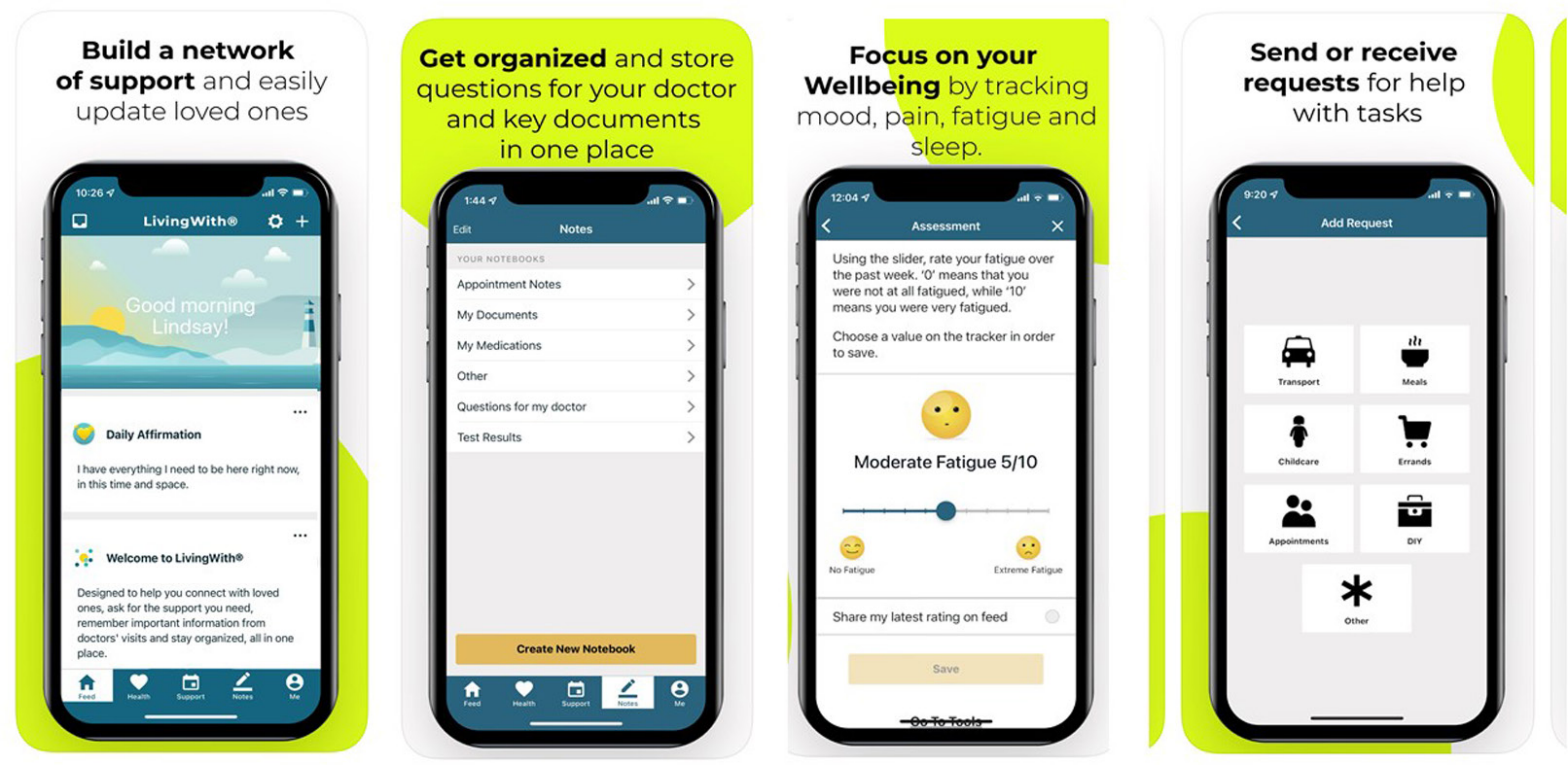
Screenshots of the LivingWith® app

Patients randomized to the intervention arm were asked to download the LivingWith® app, which is available free of cost in the Apple App store and the Google Play store. Participants were asked to enter a referral code to verify their use of the app and use it weekly for 3 months. The LivingWith® app was designed to help individuals manage life with cancer. Key app functions include: “Dashboard” to manage and view appointments and important dates; “Well‐being” to track health data from connected wearables and other health apps, and track symptoms like pain, fatigue, and sleep; “Me” to store personal health‐related documents, such as insurance cards, prescriptions, and other documents; “Health notes” to take notes and recordings from visits and pictures to support treatment; “My circle” to maintain a network of friends and families to share health updates and request emotional and logistical support; and “Resources” which provides positive affirmations, educational resources, and local event information. Select screenshots of the LivingWith® app are provided in Figure 1.

### 2.4 Study measures

All consented participants completed a survey at enrollment and at 3 months. The key study endpoints were self‐reported quality of life, health care utilization, and usability of the app (intervention group only).

#### 2.4.1 Quality of life

Two validated instruments were used to assess quality of life: the Functional Assessment of Cancer Therapy‐General (FACT‐G)^11^ and the Short‐Form Health Survey (SF‐12).^12^ Composite scores were calculated based on the user manual, which included: a composite score (27 items, range 0–108) from the FACT‐G instrument; and physical and mental component scores (each with a mean of 50 and standard deviation of 10 in the general United States population) from the SF‐12 instrument. In general, a higher score is more favorable.

#### 2.4.2 Health care utilization

Survey questions about health care utilization were adapted from validated items available through the National Health Interview Survey. They included questions about the number of times an individual received the following services in the preceding 3 months: (1) office visit, (2) visit with a mental health professional, (3) emergency room, urgent care, same‐day appointment, or walk‐in clinic visit, and (4) virtual care (phone calls, emails, or web portal messages).

#### 2.4.3 App use

We measured the numbers of logins among intervention participants using Pfizer analytics tools. In the follow‐up survey, intervention group participants were asked to rate the usefulness of the app overall, and of individual function on a 1 to 5 scale (1 = Never used, 2 = Not at all useful, to 5 = Very Useful). Structured questions about specific reasons they used and did not use the app were asked using a 1 to 4 scale (1 = Strongly Disagree, to 4 = Strongly Agree). The survey also allowed for free‐text feedback from participants regarding the reasons they used the app and concerns with using the app.

#### 2.4.4 Patient characteristics

We collected patients' age at enrollment, gender, race, education, income, marital status, smoking status, cancer types, and cancer stage at diagnosis.

### 2.5 Statistical analyses

Baseline characteristics were summarized by study arms using frequency and percentage for categorical measures and mean and standard deviation for continuous measures. Among the intervention arm, we also compared demographic and clinical characteristics stratified by whether the individual reported having used the app. Changes from baseline to the 3 month follow‐up for each outcome were constructed and compared between study arms. Student *t*‐test and Chi‐square test were used to compare continuous measures and categorical measures respectively by subgroups. Multivariable linear regression was used to examine changes in study outcomes by study arm adjusting for their baseline value. Thematic analyses were used to evaluate free‐text survey questions on app usability. We used SAS Software (SAS Institute Inc.) for our statistical analyses.

## 3 RESULTS

**FIGURE 2:**
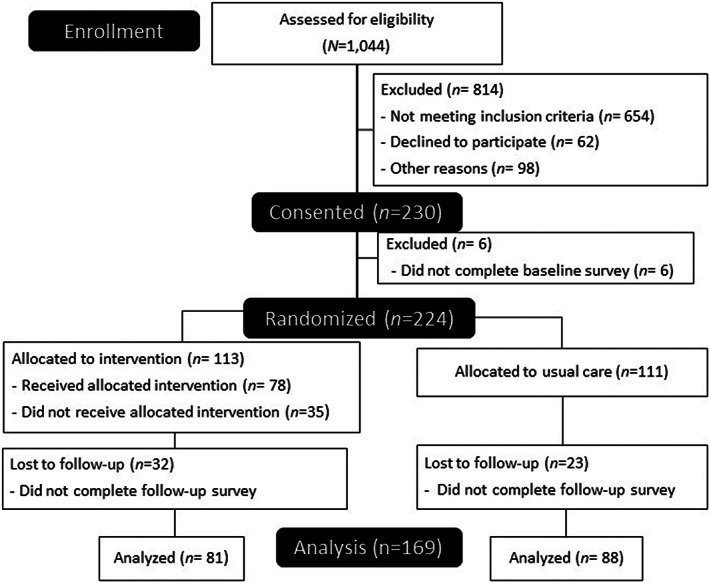
Participant flow chart

Overall, 240 patients initiating chemotherapy treatment at three WCCRI locations consented to participate in this trial from June 2020 to June 2021. A total of 224 participants completed the baseline survey and were randomized into the intervention (*n* = 113) and control arms (*n* = 111). The attrition rate was 28.3% for the intervention group and 20.7% for the control group. The final analysis included 169 patients who completed both the baseline and 3‐month follow‐up surveys (Figure 2).

The mean age was 56.3 years (SD = 12.4), 68.6% were female, 60.9% were White, and 35.5% were Black. The most common cancer among our cohort was breast cancer (*n* = 65, 38.5%), followed by hematologic cancer (*n* = 24, 14.2%), and gastrointestinal cancer (*n* = 21, 12.4%). Most participants had more than high school education (*n* = 135, 79.9%) and about half of the participants had a household income of $60,000 or more (*n* = 85, 50.3%). See Table 1 for details.

**TABLE 1:** Characteristics by study arms

	Patients, No. (%)		
	Intervention (*n* = 81)	Control (*n* = 88)	Total (*n* = 169)
Age, years			
Mean (STD)	56.0 (12.7)	56.6 (12.1)	56.3 (12.4)
Gender			
Female	53 (65.4)	63 (71.6)	116 (68.6)
Race			
Black	30 (37.0)	30 (34.1)	60 (35.5)
White	48 (59.3)	55 (62.5)	103 (60.9)
Other	0 (0.0)	1 (1.0)	1 (0.6)
Education			
More than high school	63 (77.8)	72 (81.8)	135 (79.9)
Income			
$60,000 or more	44 (54.3)	41 (46.1)	85 (50.3)
Married	58 (71.6)	55 (62.5)	113 (66.9)
Current smoker	10 (12.3)	11 (12.5)	21 (12.4)
Cancer type			
Breast	29 (35.8)	36 (40.9)	65 (38.5)
Hematologic/blood	9 (11.1)	15 (17.0)	24 (14.2)
Digestive/gastrointestinal	11 (13.6)	10 (11.4)	21 (12.4)
Gynecologic	7 (8.6)	8 (9.1)	15 (8.9)
Respiratory/thoracic	6 (7.4)	6 (6.8)	12 (7.1)
Other^a^	16 (19.8)	11 (12.5)	27 (16.0)
Cancer stage			
Stage 0–II	20 (24.7)	35 (39.8)	55 (32.5)
Stage III	20 (24.7)	18 (20.5)	38 (22.5)
Stage IV	11 (13.6)	17 (19.3)	28 (16.6)
Unknown	30 (37.0)	18 (20.5)	48 (28.4)

*Note*: Number of missing values not shown and ranged from 3 (1.8%) for education to 16 (9.5%) for smoking status.

^a^ Other cancer types include brain, colorectal, endocrine/neuroendocrine, genitourinary, germ cell, head and neck, neurologic, pineal, rectal, skin, and testicular cancers.

### 3.1 App use

**FIGURE 3:**
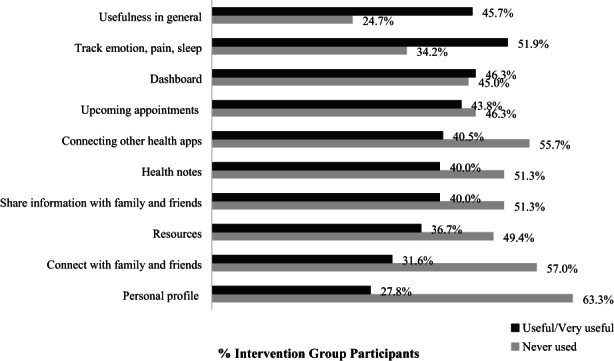
Responses to App usefulness overall and by function among intervention participants (*n* = 81). Number of missing values not shown and ranged from 0 to 2 (0%–2.5%). Percent of “Useful” or “Very useful” responses combined was calculated among those who did not report “Never used.”

Among intervention group participants, most (75.3%) reported using the app (Figure 3). Demographic and clinical characteristics were statistically similar between intervention arm participants who reported having used or never using the app (eTable S1). Notably, 80.0% of Black participants versus 70.8% of White participants, and 81.8% of those with household income less than $60,000 versus 72.7% for those with income over $60,000 reported using the app. The mean age among app users was 55.0 versus 59.0 for nonusers. The mean number of logins was 11.7 times (SD = 15.2) during the 3‐month intervention (range from 0 to 79). Among participants who had any app use, the mean number of logins was 15.6 times (SD = 15.7).

Patients' reported usefulness varied by distinct functions of the app. Overall, among those who used the app, 45.7% reported the app useful. The most used app function was tracking emotion, pain, and sleep (65.8%), and among those who used this function, more than half found it useful (51.9%). The least used app function was creating a personal profile for their documents, health insurance information and prescription (63.3% reported that they never used it), and only 27.8% of those who used this function found it useful. Other app functions, such as dashboard and upcoming appointment reminders, were more commonly used and rated as useful by 46.3% and 43.8% of users (Figure 3).

**FIGURE 4:**
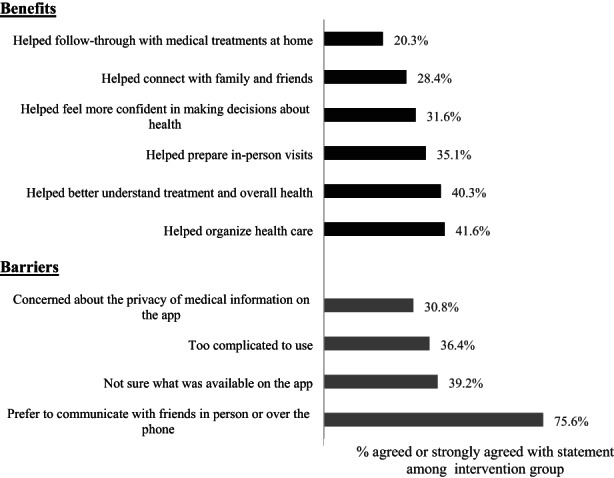
Perceived benefits and barriers for using App among intervention participants (*n* = 81). Number of missing responses for each question ranged from 2 to 7 (2%–9%). Percent of “Agree” or “Strongly Agree” responses combined in response to potential reasons for using and rarely using the app.

Among specific reasons for using the app, over 40% of the participants agreed that the app helped them better understand their treatment and overall health, and to organize health care including appointments, medications, and information related to the treatment. On the other hand, over 75% of the participants reported that a barrier to using the app was a personal preference for communicating with friends and family directly in person or over the phone instead of with an app. About a third of the participants cited the complexity of the app (36.4%) and privacy concerns (30.8%) as barriers to using the app (Figure 4).

Among a total of 42 free‐text responses for questions about the reasons and impacts of using the app, about a quarter mentioned that using it to track appointments and notes (26.8%) and track their daily symptoms and moods (24.4%) was beneficial. For example, one participant noted that: “The app helped me to keep track of my symptoms and allowed me to share with my doctor on my visits.” Another participant responded that the app: “…helped me to see how many bad days against good days.” Respondents (11.9%) specifically pointed out that the app provided them with motivation. One participant noted that: “The diary and the appointment notes are my favorite features in the app and the daily affirmations.” Some (9.5%) reported that the app was convenient to use. One participant wrote: “My husband could get my phone and pull up all my meds when I wasn't feeling like taking them during my chemo.”

Several participants (39.9%) reported not using the app due to their busy schedules, use of other similar apps, not being an app user in general, and some concerns with the usability of the app. For example, a participant noted “It would be helpful for me and would help my friend and family understand and stay updated. I was unable to utilize the app in that manner because some people were concerned about downloading the app.” Another participant wrote: “I work full time from home. The additional effort required for inputting all that data into the app was not something that I wanted to do. Just didn't have the energy.”

### 3.2 Health care utilization

**FIGURE 5:**
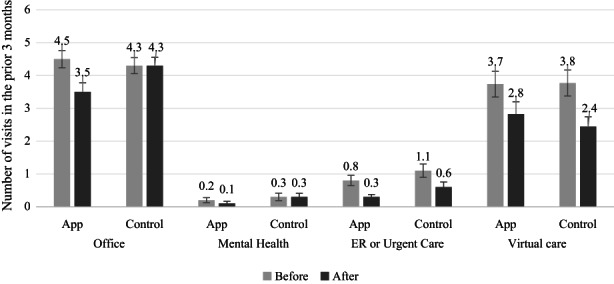
Changes in health care utilization before and after intervention. Number of missing responses for each question ranged from two for office visits at baseline (1.2%) to six for mental health visits at baseline and 3‐month follow‐up (3.6%).

At baseline, participants reported a similar level of utilization across two study arms. On average, they had about one visit to the emergency room, urgent care, same‐day appointment, or walk‐in clinic (0.8 vs. 1.1, *p* = 0.22), and less than one visit with a mental health professional (0.2 vs. 0.3, *p* = 0.61) in intervention and control groups in the prior 3 months, respectively. Participants had over four scheduled office visits (4.5 vs. 4.3, *p* = 0.72) and over three virtual care encounters in the prior 3 months (3.7 vs. 3.8, *p* = 0.95, Figure 5, eTable S2).

At 3‐month follow‐up survey, overall utilization was lower among intervention group participants than at baseline. Patients assigned to the intervention group reported having fewer office visits (3.5 vs. 4.3, *p* = 0.046) and visits with a mental health professional (0.1 vs. 0.3, *p* = 0.064) at 3‐month follow‐up compared with those in the control group. Adjusted results show 0.74 fewer office visits (*p* = 0.043) and 0.24 fewer visits with a mental health professional (*p* = 0.061) among intervention participants relative to controls (Table 2). Utilization for other services was similar across the two arms. For example, the average number of emergency visits, urgent care, same‐day appointments, or walk‐in clinic visit was 0.3 vs. 0.6 (*p* = 0.11), and the average number of phone calls, emails, or web portal messages with providers was 2.8 vs. 2.4 (*p* = 0.42) for the intervention and control groups, respectively (Figure 5, eTable S2).

**TABLE 2:** Adjusted changes in study outcomes comparing intervention and control groups

	Coefficient (95% CI)	*p‐*Value
Quality of life composite scores		
Quality of life	0.36 (−3.42, 4.14)	0.850
Physical health	−0.40 (−3.04, 2.23)	0.762
Mental health	0.04 (−2.93, 3.00)	0.981
Health care utilization		
Office visit	−0.74 (−1.45, −0.03)	0.043
Visit with a mental health professional	−0.24 (−0.49, 0.01)	0.061
Emergency room or urgent care	−0.19 (−0.51, 0.13)	0.250
Virtual care	0.45 (−0.44, 1.34)	0.320

*Note*: Models included study arm and baseline score of each outcome.

### 3.3 Quality of life

At baseline, participants in both arms reported similar quality of life across all scales (eTable S2). As expected for patients undergoing chemotherapy treatment, participants experienced worsening physical health in both arms. The mental health composite scores slightly increased (i.e., improved) from baseline. There were no statistically significant differences in these changes by study arm (Table 2).

## 4 DISCUSSIONS

This study used a randomized controlled trial to evaluate the efficacy and usability of an app (LivingWith®) that provides self‐management support for patients with cancer undergoing chemotherapy on health care utilization and quality of life. The findings demonstrate a positive impact of the intervention in reducing the number of office visits utilized and an encouraging trend in fewer visits with a mental health provider. There were no significant changes by study groups in quality of life, use of virtual care, or emergency room and urgent care visits.

Patients with cancer undergoing chemotherapy must manage an overwhelming amount of information to coordinate their treatment and related activities. Comprehensive coordinating efforts are needed to manage complex schedules and side‐effects of chemotherapy treatments. Our findings showed similar reported app use across sociodemographic subgroups, with slightly higher use among participants who identified as Black or had lower incomes (eTable S1). This suggests that the increasing availability of smartphones across socioeconomic groups and regions provides an opportunity to develop digital solutions to reduce health disparities through increasing access to educational resources and symptom monitoring and management.^13^

Apps can be leveraged by patients to support their efforts to manage their health, coordinate appointments, and keep track of treatment notes and questions.^1^ Although hundreds of health apps are available for patients with cancer, few provide support for self‐management activities, and even fewer have been rigorously evaluated.^7^, ^8^, ^9^, ^10^, ^14^ Emerging evidence suggests that health apps can be used by various population groups and may support improvements in pain and fatigue.^10^ In our study, the two study groups did not report differences in quality of life, physical health, or mental health. This may be due to the relatively short follow‐up period while patients are still in the midst of completing chemotherapy, which can impair daily function and worsen quality of life. Future studies should examine the impact of the intervention on patients in different phases of their treatment starting with the initial diagnosis, and on longer‐term quality of life after they complete their active cancer treatments.

Most intervention participants reported using the app and used it weekly during the 3‐month intervention. Notably, nearly a quarter of intervention participants who consented to participating in this app‐based study reported not even trying the app. Common barriers to using the app included personal preference to communicate with friends and family directly, usability challenges, and privacy concerns. Similarly, previous studies have found that usability was a challenge, especially for a diverse group of patients.^15^, ^16^ Satisfaction with the app was mixed with fewer than half of those using it reporting it as useful. The functions most reported as useful included tracking health, reviewing the dashboard, and upcoming appointments. The functions least commonly reported as useful included setting up a personal profile, connecting with friends and family, and connecting with other health apps. The app was designed by Pfizer to provide comprehensive support for people impacted by cancer. Providing multiple functions to support an array of self‐management activities and education may have added to the complexity of using the app. Still, developers are continuously reviewing and updating the app to improve usability. Even with improved usability, it is possible that some individuals will be more open to using technology and apps to support their health than others. The variability in personal preference highlights the importance of not relying exclusively on app‐based interventions to support self‐management activities and health behavior changes.

This is the first prospectively randomized trial of an app designed to support individuals dealing with cancer with tools to support self‐management activities that shows fewer office visits and no adverse impact on quality of life or urgent care among intervention participants relative to controls. Notably, adjusted results showed a larger reduction in health care utilization for all in‐person services (office visits, mental health, emergency or urgent care) among the intervention group relative to controls, however, the difference only reached statistical significance for office visits (Table 2). The app provided several functions to support patients and their informal caregivers to coordinate activities and track health and information during treatment. Although more research is needed to understand the mechanism for app participants to require fewer visits, it is possible that using the app to manage and track information resulted in improved self‐management and less need for visits. In fact, among app users, many reported that the app helped them to better understand their treatment plan. Despite having fewer office visits, intervention group participants reported similar levels of quality of life and urgent and emergency care. This is encouraging and implies that fewer visits were not associated with adverse outcomes for patients.

Despite its strengths, including the rigorous randomized study design, the novelty of the research, and the diverse sample of patients, this study has limitations. First, participants were recruited from a single cancer center located in the mid‐South region of the United States and the study was limited to English speakers. Findings may not be generalizable to other regions of the country, other cancer centers, and non‐English speakers. Second, intervention group participants were asked to download the study app and use it weekly when consenting to participate in the study, but app use was not enforced, and nearly 1 in 4 reported not using the app. Moreover, it's possible that participants in both groups used other apps to support their health during the study. Lastly, the study only followed participants for a short time, 3 months, and relied on self‐reported data for study outcomes.

## 5 CONCLUSION

In conclusion, in a randomized controlled trial, patients undergoing chemotherapy randomized to use an app to support self‐management activities reported having fewer office visits during the 3‐month intervention compared to controls, with no adverse effects or changes in other study outcomes. Although there are hundreds of health apps available to support patients undergoing cancer treatment, few have been rigorously evaluated. This study is the first to show that an app designed to support self‐management functions helped patients better understand and coordinate their treatment plan resulting in fewer office visits. Still, nearly a quarter of patients randomized to the intervention arm reported never using the app and among app users, fewer than half found it useful. Barriers to using the app included personal preference, privacy concerns, and usability challenges. To address these challenges, future studies should focus efforts to improve user‐centered design and test strategies to increase awareness and willingness to use apps for heath management. Nonetheless, it is important to keep in mind that not all patients will want to use apps to support their care. More research is needed to understand the mechanism for app use to reduce the need for office visits and its impact on longer‐term health outcomes.

## AUTHOR CONTRIBUTIONS

**Ilana Graetz:** Conceptualization (lead); formal analysis (equal); funding acquisition (equal); investigation (equal); methodology (equal); writing – original draft (lead); writing – review and editing (lead). **Xin Hu:** Formal analysis (lead); methodology (equal); writing – review and editing (equal). **Andrea N. Curry:** Data curation (lead); project administration (lead); writing – review and editing (equal). **Andrew Robles:** Data curation (equal); project administration (equal); writing – review and editing (equal). **Gregory A. Vidal:** Project administration (equal); supervision (equal); writing – review and editing (equal). **Lee S. Schwartzberg:** Conceptualization (equal); funding acquisition (equal); investigation (equal); methodology (equal); supervision (equal); writing – review and editing (equal).

## CONFLICT OF INTEREST

Dr. Vidal reported receiving personal fees from Roche/Genentech, Novartis, Eli Lilly, Immunometric, Puma, Pfizer, AstraZeneca, Biotheranautics, Daiichi Sankyo, Vector Oncology and research funding from Roche/Genentech, Puma, Celcuity, Merck, BMS, Eli Lilly, GTx Inc., AstraZeneca, Pfizer, Immunomedics, Tesaro, Halozyme, and ownership of Oncodisc. Dr. Schwartzberg reported receiving personal fees from Amgen, Pfizer, Helsinn, Genentech, Genomic Health, BMS, Myriad, AstraZeneca, Bayer, Spectrum, Napo and research support from Amgen, Pfizer. Dr. Graetz received research support from Pfizer. Ms. Hu received a dissertation grant from PhRMA Foundation. No other disclosures were reported.

## Supporting information


Appendix S1

## Data Availability

The data are not publicly available due to privacy or ethical restrictions.
