# Supplementary material for: Mobile application to support oncology patients during treatment on patient outcomes: Evidence from a randomized controlled trial
Source: Cancer Med. 2022 Oct 18;12(5):6190–9. doi: 10.1002/cam4.5351 (PMC10028030; doi:10.1002/cam4.5351)
Supplement: Supplementary file 1 — Appendix S1 [file CAM4-12-6190-s001.docx]

**Appendix**

**eTable 1. Participant characteristics by reported app use among the intervention group (n=81)**

|  | **Patients, No. (Row %)** | | |
| --- | --- | --- | --- |
|  | **Ever used (n=61)** | **Never used**  **(n=20)** | **P-value**^1^ |
| Age, y**:** Mean (STD) | 55.0 (13.2) | 59.0 (11.1) | 0.24 |
| Gender: Female | 41 (77.4) | 12 (22.6) | 0.38 |
| Race: Black | 24 (80.0) | 6 (20.0) | 0.37 |
| White | 34 (70.8) | 14 (29.2) |  |
| Education: High school or lower | 10 (62.5) | 6 (37.5) | 0.21 |
| More than high school | 49 (77.8) | 14 (22.2) |  |
| Income: Less than $60,000 | 27 (81.8) | 6 (18.2) | 0.35 |
| $60,000+ | 32 (72.7) | 12 (27.3) |  |
| Marital status: Married | 41 (70.7) | 17 (29.3) | 0.29 |
| Single/Divorced/Widowed | 15 (83.3) | 3 (16.7) |  |
| Smoking status: Current smoker | 7 (70.0) | 3 (30.0) | 0.74 |
| Former/Never smoker | 51 (75.0) | 17 (25.0) |  |
| Cancer Type: Breast | 22 (75.9) | 7 (24.1) | 0.77 |
| Hematologic/Blood | 10 (90.9) | 1 (9.1) |  |
| Digestive/Gastrointestinal | 5 (71.4) | 2 (28.6) |  |
| Gynecologic | 6 (66.7) | 3 (33.3) |  |
| Respiratory/Thoracic | 13 (68.4) | 6 (31.6) |  |
| Other^2^ | 5 (83.3) | 1 (16.7) |  |
| Cancer Stage: 0-II | 10 (83.3) | 2 (16.7) | 0.17 |
| III | 4 (50.0) | 4 (50.0) |  |
| IV | 13 (65.0) | 7 (35.0) |  |

*Notes:* Number of missing values not shown and ranged from 2 (2.5%) for education to 11 (13.6%) for cancer stage at diagnosis. ^1^Student t-test used to compare means and Chi-square test used to compare percentages by study arms.^2^Other cancer types include brain, colorectal, endocrine/neuroendocrine, genitourinary, germ cell, head and neck, neurologic, pineal, rectal, skin, and testicular cancers.

**eTable 2. Quality of Life and Health care Utilization by Study Arms**

|  |  | **Intervention (n=81)** | **Control (n=88)** | **P-value**^1^ |
| --- | --- | --- | --- | --- |
|  | **N Missing** | **Mean (STD)** | **Mean (STD)** |  |
| **Quality of life Composite Score** |  |  |  |  |
| Baseline | 2 | 76.1 (18.3) | 74.3 (16.5) | .52 |
| 3-month Follow-up | 2 | 78.2 (18.7) | 76.2 (17.9) | .48 |
| Change | 4 | 1.9 (12.7) | 1.9 (12.8) | .99 |
| **Physical Health Composite Score** |  |  |  |  |
| Baseline | 7 | 38.0 (9.5) | 38.7 (11.3) | .70 |
| 3-month Follow-up | 17 | 37.3 (10.5) | 37.4 (10.7) | .96 |
| Change | 20 | -1.4 (9.1) | -0.9 (8.7) | .72 |
| **Mental Health Composite Score** |  |  |  |  |
| Baseline | 7 | 48.9 (10.9) | 45.8 (11.2) | .079 |
| 3-month Follow-up | 17 | 49.3 (11.1) | 47.9 (11.0) | .43 |
| Change | 20 | 0.7 (9.1) | 2.0 (11.2) | .45 |
| **Health Care Utilization** |  |  |  |  |
| **A scheduled visit to a doctor’s office or clinic** | | | | |
| Baseline | 2 | 4.5 (2.4) | 4.3 (2.3) | .72 |
| 3-month Follow-up | 4 | 3.5 (2.4) | 4.3 (2.4) | .046 |
| Change | 6 | -0.9 (2.8) | -0.1 (2.8) | .078 |
| **Visit with a mental health professional (e.g. psychologist, psychiatrist)** | | | | |
| Baseline | 6 | 0.2 (0.7) | 0.3 (1.1) | .61 |
| 3-month Follow-up | 6 | 0.1 (0.6) | 0.3 (1.0) | .064 |
| Change | 10 | -0.1 (0.7) | 0.1 (1.2) | .17 |
| **Emergency room visit, urgent care, same day appointment, or walk-in clinic visit** | | | | |
| Baseline | 2 | 0.8 (1.4) | 1.1 (1.9) | .22 |
| 3-month Follow-up | 3 | 0.3 (0.7) | 0.6 (1.4) | .11 |
| Change | 5 | -0.4 (1.5) | -0.5 (1.7) | .85 |
| **Communicated with a healthcare provider on the phone, by email, or through a web portal** | | | | |
| Baseline | 2 | 3.7 (3.5) | 3.8 (3.7) | .95 |
| 3-month Follow-up | 3 | 2.8 (3.3) | 2.4 (2.8) | .42 |
| Change | 5 | -0.9 (3.7) | -1.4 (4.0) | .39 |

^1^ Student t-test used to compare means by study arms.
